# Implicit learning and individual differences in speech recognition: an exploratory study

**DOI:** 10.3389/fpsyg.2023.1238823

**Published:** 2023-09-07

**Authors:** Ranin Khayr, Hanin Karawani, Karen Banai

**Affiliations:** Department of Communication Sciences and Disorders, Faculty of Social Welfare and Health Sciences, University of Haifa, Haifa, Israel

**Keywords:** implicit learning, speech recognition, individual differences, perceptual learning, statistical learning, incidental learning

## Abstract

Individual differences in speech recognition in challenging listening environments are pronounced. Studies suggest that implicit learning is one variable that may contribute to this variability. Here, we explored the unique contributions of three indices of implicit learning to individual differences in the recognition of challenging speech. To this end, we assessed three indices of implicit learning (perceptual, statistical, and incidental), three types of challenging speech (natural fast, vocoded, and speech in noise), and cognitive factors associated with speech recognition (vocabulary, working memory, and attention) in a group of 51 young adults. Speech recognition was modeled as a function of the cognitive factors and learning, and the unique contribution of each index of learning was statistically isolated. The three indices of learning were uncorrelated. Whereas all indices of learning had unique contributions to the recognition of natural-fast speech, only statistical learning had a unique contribution to the recognition of speech in noise and vocoded speech. These data suggest that although implicit learning may contribute to the recognition of challenging speech, the contribution may depend on the type of speech challenge and on the learning task.

## Introduction

Human listeners vary in their ability to recognize speech, especially in challenging listening situations (e.g., noisy backgrounds, rapid speech rates, noise vocoding). In such situations, some listeners struggle more than others to recognize speech. These individual differences are partially explained by sensory, cognitive and linguistic factors (Gordon-Salant and Fitzgibbons, 1997; Akeroyd, 2008; Stenfelt and Rönnberg, 2009; Adnak and Janse, 2010; Benichov et al., 2012; Janse and Adnak, 2012; Mattys et al., 2012; Tamati et al., 2013; Fullgrabe et al., 2014; Heald and Nusbaum, 2014; Banks et al., 2015; Bent et al., 2016; Carbonell, 2017; McLaughlin et al., 2018). Another factor involved in speech recognition is implicit learning (Adnak and Janse, 2009; Conway et al., 2010; Vlahou et al., 2012). Broadly defined, implicit learning occurs without awareness of what is being learned and without a clear intention to learn and refers to all learning experiences that result in the formation of non-declarative memories (Squire and Dede, 2015; Pisoni and McLennan, 2016). However, its contribution to individual differences in speech recognition and how learning may interact with the contributions of other sensory and cognitive factors is not well understood (Banai and Lavie, 2020; Heffner and Myers, 2021). Our goal here was to explore the associations between different indices of implicit learning - perceptual, statistical, and incidental - and the recognition of challenging speech (fast, vocoded and speech in noise). Speech recognition in this study refers to the accuracy (proportion correct) of word identification in the sentences we used. Data were modeled to determine whether each index of implicit learning contributed to the recognition of each type of challenging speech after accounting for the potential contributions of age and cognition (vocabulary, memory, and attention).

Speech recognition relies on a dynamic interplay of sensory and higher-level cognitive and linguistic processes (Mattys et al., 2009; Fullgrabe et al., 2014; Rönnberg et al., 2019). Therefore, the relative contributions of different processes to speech recognition may change between different listening situations and different types of speech (Gordon-Salant and Fitzgibbons, 1997; Fullgrabe et al., 2014; Heald and Nusbaum, 2014; Bent et al., 2016; McLaughlin et al., 2018; Rönnberg et al., 2019). According to interactive models such as the Ease of Language Understanding model (ELU, Rönnberg et al., 2019, 2021), in ideal conditions speech recognition is automatic and the incoming acoustic signal can be implicitly matched to existing linguistic representations. Under more challenging conditions (e.g., degraded speech, background noise), there is ambiguity in how the incoming acoustic signal matches existing representations, rendering automatic processing insufficient. In such conditions, higher-level cognitive processes (such as working memory and attention), contextual and linguistic knowledge and semantic information are recruited to resolve the mismatch and afford recognition. For example, there is evidence that higher working memory capacity is associated with more accurate speech recognition, especially under noisy conditions (Janse and Adank, 2012). Likewise, cognitive flexibility predicted differences in comprehension of a novel accent by younger and older adults (Adnak and Janse, 2010). In the current study we used natural-fast speech, speech in noise and vocoded speech to assess speech recognition under challenging conditions. Whereas fast speech yields a temporal challenge similar to that induced by the time-compressed speech learning task, vocoded speech creates a spectral challenge (the spectrum of the stimuli is impoverished), while speech in noise represents a challenge due to the masking of the target speech by noise created by additional speakers.

There are multiple demonstrations that under challenging conditions speech recognition can improve rapidly, but unintentionally, consistent with a contribution of implicit learning (Adnak and Janse, 2009; Vlahou et al., 2012). For example, very brief exposure to time-compressed speech in an unfamiliar language (Catalan) resulted in measurable improvements in the recognition of time-compressed speech in a familiar language (Spanish) (Pallier et al., 1998). Therefore, we assume that implicit learning is involved in the recognition of different types of degraded and challenging speech (Banai et al., 2022). However, the literature describes different indices of implicit learning – perceptual, statistical, and incidental learning (described in the following paragraphs), each studied with different paradigms, and it is not clear whether they share the same underlying mechanism. If different indices of learning reflect the same underlying capacity, this capacity could be utilized across a range of challenging conditions when the incoming signal does not match existing lexical representations automatically. Recent studies on visual (Yang et al., 2020; Dale et al., 2021) and auditory-speech learning suggest that at least across some tasks, learning could rely on a shared underlying capacity. However, the consequences for individual differences in speech recognition are not well understood. In the following paragraphs, we describe the different indices of learning used in our study and what is known about their associations with speech recognition.

Perceptual learning is defined as improvements in the ability to process stimulus-related information following experience or practice (Green et al., 2018). Experience with challenging or unusual speech often leads to perceptual learning (Adnak and Janse, 2009; Samuel and Kraljic, 2009; Baese-Berk et al., 2013; Banai and Lavner, 2014). For example, the recognition of both accented and rapid speech improves within minutes of exposure due to perceptual learning (Samuel and Kraljic, 2009). Perceptual learning is implicit because while listeners might be aware that they are improving, they find it hard to verbalize what they learned (Fahle et al., 2002). Rapid perceptual learning is linked to individual differences in speech recognition even across conditions (see Banai et al., 2022). For example, Karawani and Banai (2017) elicited rapid learning of speech in noise in older adults using a passage comprehension task. Participants were also tested on two speech-in-noise tasks with which they had no prior experience: discrimination of pseudowords and sentence verification. The magnitude of rapid learning on the passages task explained more than 30% of the variance in performance on the other tasks. Rapid perceptual learning was still associated with individual differences in speech recognition in independent tasks even after considering the contributions of cognition and hearing (Rotman et al., 2020; Banai et al., 2022). As in previous studies, we evaluated perceptual learning with time-compressed sentences.

Statistical learning is another index of implicit learning that supports learning of patterns and sequences. It reflects the ability to learn to extract regularities across stimuli by detecting the probabilities with which properties co-occur with no explicit awareness of those regularities and with no instruction to learn (Misyak and Christiansen, 2012). Statistical learning tends to be studied with stimuli in sequences such as the serial reaction time paradigm where participants are passively exposed to strings generated by an artificial grammar or continuous sequences of nonwords from an artificial lexicon. Following this brief exposure participants can incidentally acquire knowledge about the predictive relations embedded within the stimuli (Gomez and Gerken, 2000; Saffran, 2003). Exposure to positive instances facilitates learning, without engaging in analytical processes or explicit hypothesis testing strategies (Banai and Lavie, 2020). Statistical learning occurs beyond individual stimuli and plays a role in language acquisition and processing (Saffran et al., 1996; Kuhl, 2004; Conway and Christiansen, 2005, 2009; Turk-Browne et al., 2005; Conway et al., 2007; Mirman et al., 2008; Kidd, 2012; Kidd and Arciuli, 2016). Statistical learning is associated with the acquisition of syntax (Kidd and Arciuli, 2016), word segmentation (Saffran et al., 1996), word learning (Mirman et al., 2008), and speech recognition (Conway et al., 2007; Conway and Christiansen, 2009). Recent work focused on individual differences in statistical learning and how they may associate with individual differences in speech recognition (Conway et al., 2010; Misyak et al., 2010; Misyak and Christiansen, 2012; Theodore et al., 2020). For example, Conway and colleagues (Conway et al., 2010) demonstrated that implicit statistical learning (both auditory and visual) is significantly correlated with speech recognition even after controlling for sources of variance associated with intelligence, working memory, attention, inhibition, knowledge of vocabulary and syntax. In their study statistical learning was assessed with a visual color sequence task and an auditory non-word sequence recall task in the auditory modality. In both modalities the sequences were based on an artificial grammar. Speech recognition was assessed with degraded sentences that varied in the predictability of the final word. Another study by Neger et al. (2014) demonstrated a relationship between visual statistical learning (assessed with an artificial grammar learning serial reaction time paradigm) and individual differences in the recognition of vocoded speech. Here we adopted that visual learning task (Neger et al., 2014) to further examine the association between statistical learning and the individual differences in the recognition of challenging speech.

Incidental learning (also called task irrelevant perceptual learning or TIPL) refers to improvement in the detection or discrimination of a stimulus, which is apparently not related to the task being practiced (Seitz and Watanabe, 2005). Rather, the target stimulus is displayed with no conscious effort directed at it. One way to elicit incidental learning is to present two streams of stimuli and instruct participants to attend to and respond to targets in only one of them. Learning results in relatively better recognition of non-attended items in the other stream that were delivered simultaneously with the attended items (Protopapas et al., 2017). Incidental learning is thought to play a role in different aspects of language such as language acquisition, non-native speech learning and orthographic processing (Saffran et al., 1997; Hulstijn, 2012; Protopapas et al., 2017). For example, incidental learning of orthographic forms affects both reading and spelling (Protopapas et al., 2017). Demonstrating that incidental learning contributes to speech perception, Vlahou et al. (2012) studied the recognition of a difficult non-native speech sound identification following either incidental or explicit training. Greek speakers were exposed to a difficult contrast in Hindi. The stimuli were recordings of consonant-vowel syllables with retroflex and dental unvoiced stops by a native Hindi speaker. On each trial, participants heard pairs of tokens from both categories and were unaware that in the implicit conditions, target sounds were always retroflex, and distractor sounds were always dental. Post-training, all trained groups performed better than untrained Greek speakers. However, learning was most robust following implicit training without feedback. If incidental learning involves a modality-general component, visual incidental learning should also be associated with speech recognition. Therefore, here we investigated the role of visual incidental learning in individual differences in challenging speech recognition.

As stated above, it is not clear whether the contributions of perceptual, statistical and incidental learning to speech processing reflect shared or distinct underlying mechanisms, and whether they make independent or overlapping contributions to speech recognition (Squire and Dede, 2015; Fiser and Lengyel, 2019). Learning might be shaped by the (modality) specific characteristics of the task and stimuli used to elicit learning, therefore ideally, all indices of learning should be evaluated with visual tasks. However, modality specific effects do not rule out the possibility of a general learning ability (see Yang et al., 2020). Rather, Bayesian learning approaches suggest that different indices of learning can be treated within a unified framework, especially when complex stimuli are considered (Fiser and Lengyel, 2019). To the extent that implicit learning is a general, rather than a modality specific process (see Siegelman et al., 2017; Yang et al., 2020), associations between implicit learning and speech recognition should not be limited to the auditory modality. Additionally, studies on the associations between individual differences in auditory perceptual learning and individual differences in speech recognition suggest that rapid learning is key (Karawani and Banai, 2017; Rotman et al., 2020; Banai et al., 2022), but visual perceptual learning is hard to document with such brief exposure. Therefore, in the current study we evaluated perceptual learning with the same auditory task used in the past (Rotman et al., 2020; Banai et al., 2022), whereas statistical and incidental learning were evaluated with visual tasks to quantify the possible contribution of a more general learning process (Yang et al., 2020; Dale et al., 2021). As noted above, associations between speech recognition and visual implicit and statistical learning were already documented (Conway et al., 2010; Misyak and Christiansen, 2012), consistent with the idea that the association between implicit learning and speech recognition is not confined to the auditory modality.

Against this background, we now ask whether each index of implicit learning (perceptual, incidental, and statistical learning) makes a unique contribution to the recognition of three types of challenging speech (natural fast, vocoded and speech in noise) beyond the contribution of other known cognitive factors. Based on the literature reviewed above, we hypothesize that each of the different indices of implicit learning may contribute to the recognition of challenging speech, but their contribution might change between different speech tasks. If each learning index reflects an independent learning process, each may make a unique contribution to speech recognition, but current literature is insufficient to formulate more accurate hypotheses.

## Materials and methods

### Participants

Fifty-one participants were recruited through advertisements at academic institutions and social media. One participant was excluded from the study because his performance in the statistical learning task was about 3 standard deviations greater than the average performance of the group. The remaining 50 participants (age range 19–35, *M* = 25 years, 16 men, 34 women) were naïve to the purposes of the study and (by self-report) met the following inclusion criteria: (a) High school education or higher; (b) Hebrew as first or primary language; (c) No known neurological conditions; (d) Normal hearing; (e) Normal or corrected to normal visual acuity and normal color vision. All aspects of the study were approved by the ethics committee of the Faculty of Social Welfare and Health Sciences at the University of Haifa (protocol number 419/19).

### Test battery

Participants completed a test battery that included challenging speech recognition tests, cognitive measures, an auditory speech perceptual learning task and two visual learning tasks (statistical and incidental). Participants were compensated for their time.

### Speech recognition

Stimuli were 60 different sentences in Hebrew (based on Prior and Bentin, 2006). Sentences were five to six word long in a common simple Hebrew sentence structure (subject-verb-object). Each sentence had five content words and a maximum of one function word. Half of the sentences were semantically plausible (e.g., “the municipal museum purchased an impressionistic painting”) and the other half were semantically implausible (e.g., “the comic book opened the back door’). Sentences were recorded by two women native-Hebrew speakers (talker 1 and talker 2) and sampled at 44 kHz via Audacity using a standard microphone and PC soundcard (Rotman et al., 2020). The level of the sentences was normalized after recording to the RMS amplitude was similar across all audio files containing the original sentences. This was done before manipulating the audio as described below. During testing participants were instructed to adjust the level of speech presentation to their comfortable level. This was done with a list of 3 sentences not otherwise used in the study. Afterwards, sentences were presented in sequence. After each sentence, participants were asked to repeat the sentence they have heard as accurately as possible before moving on to the next sentence. A fixed list of sentences was used for each task for all participants. Otherwise scoring might become too confusing, especially when performance is poor. The speech recognition tests lasted approximately 30 min. Overall, 60 different sentences were used for the speech recognition tasks.

Natural-fast speech (NF). Twenty different sentences recorded by talker 1 were presented. Speech rate was 215 words/min (SD = 16). To obtain the natural-fast recordings, the talker was instructed to speak as fast as she could without omitting word parts. Each sentence was recorded 3 times; the clearest version of each was selected by two native Hebrew speakers who listened to all the recordings.

Noise vocoded speech (*VS*). Twenty different sentences recorded at a natural rate of 109 words/min with an average sentence duration of 3 s, by talker 2 were vocoded with Waked et al. (2017) MATLAB algorithm. To obtain the vocoded stimuli, the sentences were band-pass filtered into six channels using fourth-order Butterworth filters. The corner frequencies covered a frequency range from 200 to 8,000 Hz. The speech envelopes were isolated by applying a second-order low-pass filter with a cutoff frequency of 32 Hz. These envelopes were utilized to modulate **noise** carriers. The stimuli were produced by summing the 6 channels into the acoustic waveform and then equating the root-mean-square energy to that of the original sentences. The decision to use 6 frequency bands was based on a previous study (Bsharat-Maalouf and Karawani, 2022).

Speech in noise (SIN). Twenty different sentences recorded by talker 2 were embedded in four-talker babble noise. The SNR was −5 dB, determined based on Rotman et al. (2020) in which the same recordings were used in a different sample of young adult. The babble noise consisted of two women and two men who read Hebrew prose. The recordings of the four talkers were mixed to a single channel after the amplitude of each was maximized to just below peak to avoid clipping. Different noise segments were used to avoid adaptation and reduce the potential effects of the unique characteristics of an individual segment.

Scoring. The number of correct words per sentence was counted for each participant and condition separately. The proportion for correctly recognized words in each sentence was used for statistical modeling.

Note that the three types of speech were not produced by the same speaker because natural-fast speech is different from vocoded speech and speech in noise. It is created by asking speakers to accelerate their speech rates as much as possible while still producing all speech segments. The maximal natural-fast speech rate of most talkers, including speaker 2, is still not very challenging to young adults with normal hearing. On the other hand, vocoded speech and speech in noise are created with signal processing and can be made more difficult by reducing the number of channels (vocoded speech) or the SNR (speech in noise). Speaker 1 is fast by nature. She is simply not up to speaking more slowly as required for the purpose of creating vocoded speech or speech in noise. It is hard to find one speaker who could speak slowly and clearly enough for speech in noise and vocoded speech, but fast enough to yield challenging natural-fast speech. In the current study, no such speaker was found, therefore, we decided to use two different talkers. Talker differences and their possible influence on the results will be discussed in the discussion section.

### Cognitive measures

Two subtests from the Wechsler Adult Intelligence Scale-III (Hebrew version) (Goodman, 2001) were administered: Digit span was used to assess *working memory*; Vocabulary was used to assess *vocabulary*. The Vocabulary subtest is expressive. It measures semantic knowledge, verbal comprehension and expression, verbal fluency and concept formation, word knowledge, and word usage (Edition, 1997). Participants were asked to define words that were auditory presented to them. The test lasted approximately 10–15 min. The working memory subtest measures short-term auditory memory and attention and it consists of two parts: Forward in which participants have to repeat increasingly longer strings of digits in the same order as presented by the examiner, and backward in which participants are asked to repeat similar sequences of digits in reverse order. The task lasted approximately 10 min. Administration and scoring followed the test manual. The standardized scores (based on the test manual) were reported for the descriptive statistics (Table 1) and the scaled raw scores were entered to the statistical analysis.

**Table 1:** Age, cognition, speech recognition and learning.

	*M*	SD	Min	Max	Median
Age (years)	25	4	19	35	24
Natural-fast speech (proportion)	0.67	0.12	0.45	0.98	0.65
Vocoded speech (proportion)	0.54	0.12	0.24	0.76	0.57
Speech in noise (proportion)	0.44	0.16	0.09	0.78	0.47
Working memory (standardized scores) *	12	3.31	5	18	13
Working memory (raw scores)	21	4.59	10	28	22
Vocabulary (standardized scores) *	12	1.84	8	16	2
Vocabulary (raw scores)	47	6.55	30	60	49
Attention (Flanker cost)	1.01	0	0.99	1.02	1.01
Perceptual learning (slope)	0.008	0.008	−0.004	0.031	0.006
Statistical learning (facilitation score)	0.004	0.12	−0.31	0.46	0.0002
Incidental learning (difference score)	0.038	0.054	−0.075	0.225	0.025

*As explained in the method section, raw scaled scores were entered to the statistical modeling.

*Selective attention* was measured using the flanker task (Eriksen and Eriksen, 1974). On each of 72 trials participants had to determine the direction of a target stimulus (an arrowhead pointing to the right or to the left). The target was flanked by four additional stimuli in one of three conditions (each presented 24 times): congruent, in which the target and flankers were all arrowheads pointing at the same direction (>>>>> or <<<<<), incongruent in which the target and flankers pointed to opposite directions (<<><< or >><>>), and a neutral condition in which the target was flanked with = signs (==>== or ==<==). Each trial started with an auditory alert (a 400 Hz pure tone) and a fixation cross that remained on the screen for 250 ms followed by the target and flankers which remained on the screen for a maximum of 1,500 ms. Participants were instructed to respond as fast and as accurately as they could (by clicking either the ‘z’ or the ‘/’ key on the keyboard). The inter-trial interval was 1,000 ms. Six practice trials were presented before the start of the test. Selective attention was quantified with the flanker cost for each participant, defined as the ratio of reaction times (log RT) in the incongruent and neutral conditions (Scharenborg et al., 2015). The task lasted approximately 5 min.

This study was conducted as part of a larger study on cochlear implant users. Therefore, the following additional cognitive measures were administered to all participants, but not otherwise analyzed or reported in this manuscript: *Matrix reasoning* (Edition, 1997), *Visual Lexical Decision Task (LDT)* (adapted from Picou et al., 2013), and the Hebrew version of the *Rey Auditory Verbal Learning Test* (Vakil et al., 2010).

### Auditory perceptual learning

Rapid perceptual learning was assessed using 30 sentences presented as time-compressed speech with speech rate of 269 words/min (SD = 17). Sentences were all different from the ones used for speech recognition tasks described above. To create time-compressed stimuli, the natural (unhurried) recordings of talker 2 were compressed with a WSOLA algorithm (Verhelst and Roelands, 1993) in MATLAB to 0.35 of the original duration of each sentence. The task was otherwise similar to the speech tasks described above. Perceptual learning was quantified by calculating the linear slope of the learning curve connecting sentence number and proportion of correctly recognized words per sentence. The task lasted approximately 12 min. The use of time-compressed speech to elicit learning might be criticized given its similarity to natural-fast speech which is one of the dependent measures. However, the two have different spectral and temporal characteristics. Whereas time-compressed speech is created by uniformly shortening all speech segments without changing the long-term spectral characteristics of the original signal, natural-fast speech is qualitatively different from the unhurried speech of the same talker and involves more coarticulation and assimilation sometimes even leading to deletion of segments (Adnak and Janse, 2009). Therefore, at similar rates, natural-fast speech is more difficult to process than time-compressed speech (Janse, 2004) and learning of time-compressed speech might thus not be simply associated with the recognition of fast speech.

### Visual statistical learning

The serial reaction time artificial grammar learning task used by Neger et al. (2014) was adapted and coded in the Visual Studio environment to assess implicit sequence learning. The stimuli consisted of eight visual shapes (triangle, hexagon, star, square, arrow, circle, heart, and cross). On each trial, participants were presented with four shapes in a 2 × 2 array on a computer screen and were instructed to click as quickly as possible, using a computer mouse, on the target shape marked with a small filled red cross. Participants were required to click on two successive targets; The second target appeared after the participant selected the first target and could be predicted according to grammatical rules (e.g., a triangle will always be followed by a star or a square, but never by a heart). Participants were not able to make errors: the experiment proceeded only if a participant clicked on the appropriate target shape. The task comprised of five practice trials followed by 20 blocks of 8 trials each, as follows: (a) 16 exposure (grammatical) blocks where all grammatical combinations were repeated once, resulting in 128 exposure trials (8 × 16); (b) 2 test blocks (ungrammatical) (c) A recovery phase consisting of two grammatical blocks. After each block, a small break was implemented to avoid fatigue effects. The task lasted approximately 20 min.

Scoring. Reaction times (RTs) were measured from target highlighting to the subsequent mouse response and used to calculate facilitation scores which served as an index of individuals’ sensitivity to implicit regularities. The facilitation score was calculated by dividing the RT to the first (unpredictable) target within a trial (which served as a baseline) by the RT to the second (predictable) target in the same trial. In other words, if participants learn to predict the second target, RTs to the second item will be faster than RTs to the first unpredictable target, resulting in a higher facilitation score (facilitation score = RT1/RT2; If RT2 is smaller, then the facilitation score is higher). On the other hand, when changing the grammatical rules in the test phase (blocks 17–18), participants are expected to have some delay in the response to the second target because the rule that they had implicitly learned throughout the exposure phase had suddenly changed, resulting in a higher RT to the second target and subsequently, lower facilitation score. Overall statistical learning was quantified by the difference in facilitation scores between the last four blocks of the exposure phase (blocks 13–16) and the subsequent ungrammatical test phase (blocks 17–18). Any drop in the facilitation score between the two phases indicates that participants were affected by the removal of the underlying regularities suggesting statistical learning.

### Visual incidental learning task

The task used by Protopapas et al. (2017) was adapted and coded in the Visual Studio environment. Stimuli were 10 pairs of Hebrew words and 270 black and white line drawings. The words and drawings constructed “word training sequences” of 10 items (two words and eight images) each. One word was defined as “target word” (non-attended) and presented in blue while the other word was defined as “non-target word” (attended) and presented in red font. Participants were instructed to carefully watch the rapidly flashing sequence and to press a button (right Ctrl on the keyboard) as soon as they see something red. The reaction time as well as the accuracy of the response (hit/miss) were recorded and saved in a designated file. As soon as target detection sequence ended, a screen displayed the question “did you see the following picture in this run”? followed by an image centered on the screen. Participants were instructed to press the right or the left Ctrl key for positive and negative response, respectively. The image was either from the training sequence or not. Each participant was required to undergo a training run that consisted of 4 blocks with 50 trials each (total of 200-word training sequences). During training, awareness to the words was minimized as participants were not required to process the words and there had been no mention of the role of the words. After completing the training run, 40 words (10 pairs that were presented throughout the practice and another 10 new pairs) were randomly presented on the screen and participants were instructed to indicate for each word whether it has been included in the training run or not by pressing a button. In both parts of the task (training run and the following word identification task) the reaction time as well as the accuracy of the response (hit/miss) were recorded and saved in a designated file. Following the definition of “incidental learning” presented in a previous section, incidental learning results in relatively better recognition of non-attended items (words defined as target words and presented in blue font) compared with the recognition of attended items (words defined as non-targets and presented in red). Learning was therefore calculated as the difference in the proportion of recognized non-attended target words and attended non-target words. Greater positive values indicate more incidental learning. The task lasted approximately 20 min.

### Study administration

Participants were tested at the Auditory Cognition Lab at the University of Haifa before the onset of the COVID-19 pandemic. After a brief explanation about the procedure, participants signed a written informed consent and completed a short background questionnaire. Participants then completed the test battery in one testing session of approximately 2 h. The order of the tests was randomly determined for each participant.

### Statistical modeling

To account for the potential contribution of each index of learning (perceptual, statistical, and incidental) to speech recognition, we modeled performance in the speech recognition tasks as a function of each index of learning as well as working memory, vocabulary, attention which were previously shown to correlate with speech recognition. A series of generalized linear mixed models was implemented using the lme4 package (Bates et al., 2014) in R (R Core Team, 2019). Random intercepts for participant and sentence were included. Based on Chen et al. (2017) and Dunn and Smyth (2018), we used binomial regressions to model proportions. Raw values of all variables were used for statistical analysis. Prior to modeling, these raw values were scaled using a designated function in R. Scaling was employed because we considered multiple variables that were measured with different scales. To isolate the unique contribution of each index of learning to speech recognition, 4 models were constructed for each index of speech recognition (natural-fast speech, vocoded speech, speech in noise). The first, “Basic” model included age, cognitive variables, and time-compressed speech (TCS) recognition, which reflects overall performance in the perceptual learning task (averaged across all time-compressed sentences). Then, in a stepwise manner, we added the different learning indices to the basic model, as follows:

1. Model 1: A “Basic” model with age, working memory, vocabulary, attention, and time-compressed speech recognition.
2. Model 2: “Basic” + perceptual learning
3. Model 3: “Basic” + perceptual learning +incidental learning
4. Model 4: “Basic” + perceptual learning + incidental learning + statistical learning.

Note that two variables related to the TCS task were included in the models. The first variable is “TCS recognition” which serves as a control variable, and it refers to the average individual performance in the TCS task. It was included to account for the inherent correlation between recognition of time-compressed-speech and other forms of challenging speech, most notably fast speech. Because the recognition of time-compressed and natural-fast speech are correlated, documenting the unique contribution of learning on time-compressed speech to natural-fast speech recognition requires that the statistical model accounts for the inherent correlation between the recognition of the two types of stimuli. The second variable is “perceptual learning” which refers to learning in the perceptual learning task and is measured by the slope or the rate of change over sentences.

Perceptual learning was included in the models first (prior to incidental or statistical learning) because its potential contribution to speech recognition was somewhat expected based on our previous studies on natural-fast speech and speech in noise (Rotman et al., 2020; Banai et al., 2022). Furthermore, given the similarities between the perceptual learning task and stimuli, and the dependent speech measures, it was more reasonable to include the other indices of learning only after accounting for this similarity.

To determine whether the more complex models capture the data better than the simpler ones, we compared for each speech task the fits of each two successive models with the anova() function. If the comparison was significant (*p* < 0.05) we determined that the more complex model fits the data better than the simpler one, and therefore that the last entered learning index has a significant contribution to performance on the modeled speech task.

## Results

### Cognition and speech recognition

**Figure 1:**
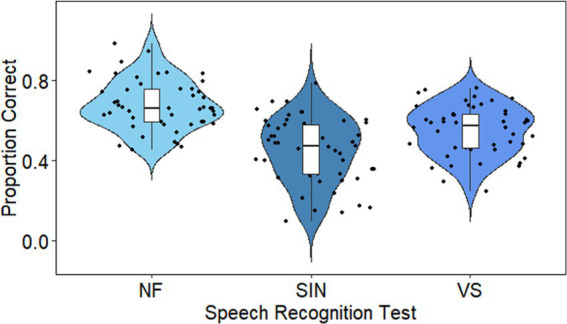
Speech recognition. Dots show individual data (averaged across sentences). Each ‘violin’ represents the distribution or response accuracies in one of the speech tests (left to right: natural fast, speech-in-noise and noise vocoded conditions). The shape of each violin represents the probability density function (PDF) of the data. A wider PDF indicates that the value occurs more frequently, and a narrower density function indicates less frequent occurrence of the value. The box plot in each violin marks the minimum, first quartile, median, third quartile and maximum.

Descriptive statistics for all variables are shown in Table 1. Speech recognition was quite variable across all three tasks. Although the goal was not to directly compare the three speech conditions, it seems that speech in noise and vocoded speech were somewhat more challenging than natural-fast speech (see Figure 1). Working memory and vocabulary scores were within the higher end of the normal range.

### Learning

**Figure 2:**
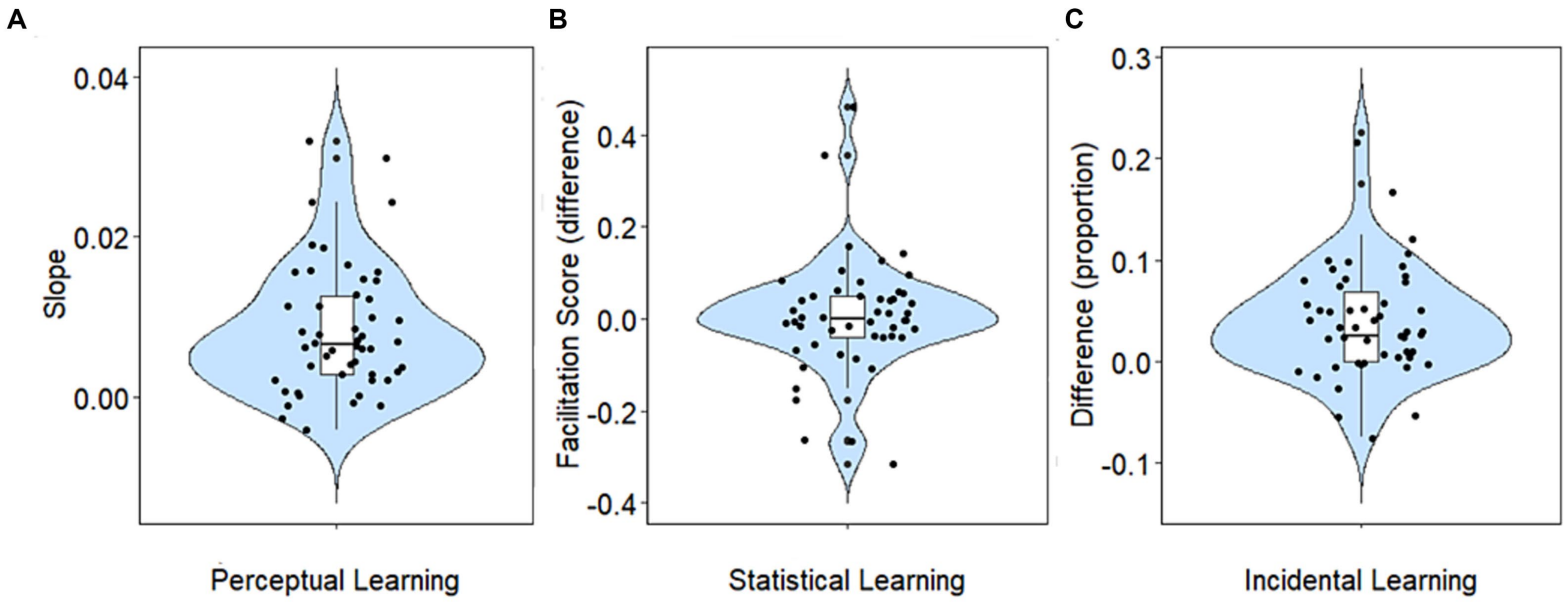
Learning. **(A)** Perceptual learning (slopes). **(B)** Statistical learning (the difference in facilitation score between the end of the exposure phase and the test phase). **(C)** Incidental learning (the difference in proportion between the recognized target versus non-target words). Each violin plot summarizes the data in a specific learning task. Across tasks, larger *y*-axis values indicate more learning than lower ones. The boxplot in each violin shows the minimum, first quartile, median, third quartile and maximum. The shape of the data in each learning task is represented by the probability density function (PDF). A wider PDF indicates that the value occurs more frequently, and a narrower density function indicates less frequent occurrence of the value.

Learning on each of the perceptual, statistical, and incidental learning tasks was quantified as explained in the methods. The distributions of the resulting learning indices are shown in Figure 2. Perceptual learning was assessed by tracking recognition accuracy over the course of 30 sentences. Subsequently, the slopes of the learning curves of individual participants were calculated as explained in the methods (see Figure 2A). Learning, indicated by a positive slope of the learning curve, was observed in 45 out of 50 participants. The mean slope was 0.008 (SD = 0.008), suggesting that the recognition improved by approximately 0.2 words/sentence.

**Figure 3:**
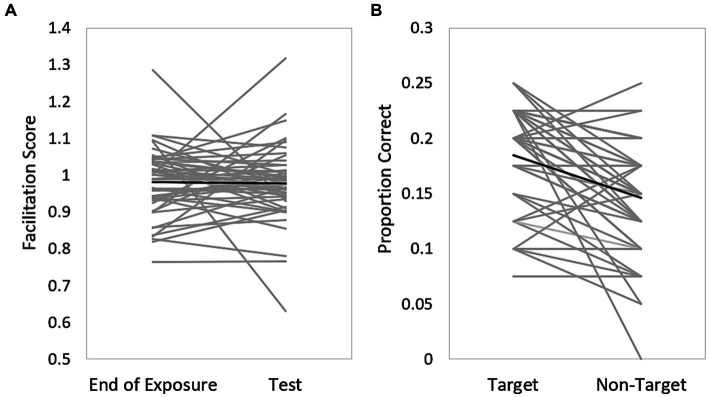
**(A)** Performance in the statistical learning task. The drop in facilitation scores from the end of the exposure phase (blocks 13–16) to the test phase (blocks 17–18) indicates learning. **(B)** Performance in the incidental learning task. Gray lines represent data from individual participants, proportion of correct identified target versus non-target words. Black line represents the averaged performance of all participants.

In the statistical learning task, a drop in facilitation score from the end of the exposure phase (blocks 13–16) to the test phase (17–18) indicates learning (see Figure 3A). The average difference in the facilitation score between the end of the exposure phase and the test phase was 0.004 (SD = 0.12). At the individual level only 25 out of 50 participants presented statistical learning. These participants implicitly learned the grammatical rules in the exposure phase, and as the rules changed in the test phase, their reaction time to the second target was longer, resulting in a decreased facilitation score.

In the incidental learning task, 33 out of 50 participants presented incidental learning as they were able to better identify non-attended target words (presented in blue font) than attended non-target words (presented in red font). Of the remaining 17 participants, 12 participants did not learn at all (equal identification of both target and non-target words), whereas 5 participants identified better attended non-target words compared to non-attended target words. Figure 3B shows the proportion of positive responses (determining that a word was present during training) for target vs. non-target words.

### Speech recognition vs. implicit learning

**Figure 4:**
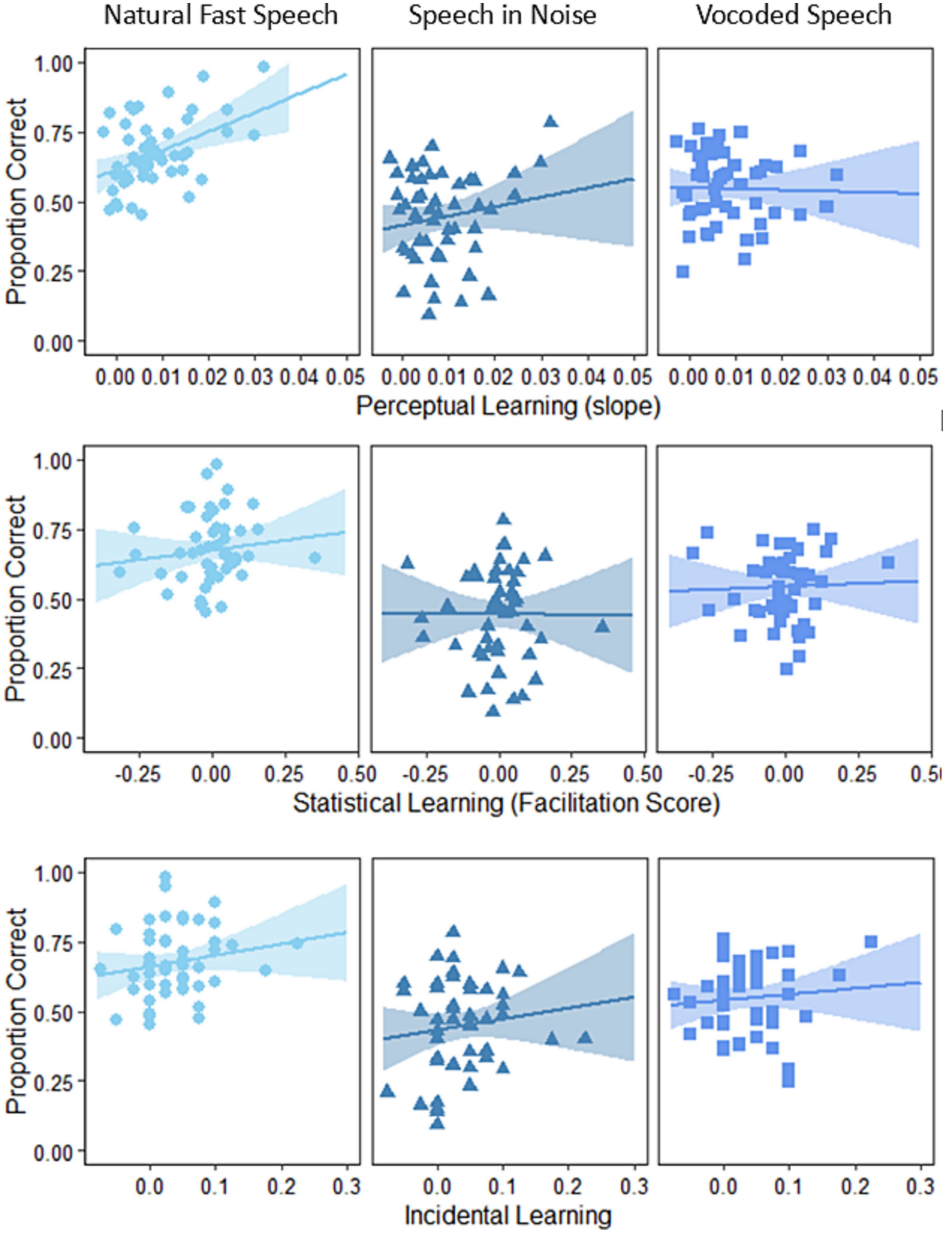
Association between implicit learning indices and the three types of challenging speech. Each row represents an implicit learning index, and each column represents a type of challenging speech.

The associations between speech recognition and implicit learning indices are presented in Figure 4 for visualization. Note though that the conclusions of this study are based on the outcomes of the statistical modeling described below.

The correlations among the different speech tasks were not high (see Table 2). Likewise, the highest correlation between learning tasks was 0.08, and the correlations between learning and the cognitive indices were also quite low (Table 2).

**Table 2:** Pearson correlations among measures of speech recognition, cognition, and learning.

	NF	VS	SIN	WM	Vocabulary	Attention	PL	SL
VS	0.35							
SIN	**0.47**	0.20						
WM	0.16	0.02	0.09					
Vocabulary	0.11	0.04	0.20	0.05				
Attention	0.04	0.05	0	0.13	−0.06			
PL	0.40	−0.05	0.11	0.19	0.13	−0.12		
SL	0.16	0.22	0.09	0.07	0.19	0.04	−0.11	
IL	0.17	0.09	0.13	−0.21	0.12	−0.01	0.08	0.03

NF, natural-fast speech; VS, vocoded speech; SIN, speech in noise; WM, working memory; PL, perceptual learning slope; SL, statistical learning; IL, incidental learning. For this sample size correlations > 0.26 are significant at *p* = 0.05. Significant correlations after correcting for multiple comparisons (*p* < 0.0014) are marked in bold.

#### Recognition of natural-fast speech

Model comparison for the recognition of natural-fast speech suggested that the addition of each type of implicit learning thus resulted in a model that better fits the data: model 2 was significantly better than model 1 (χ^2^ = 16.06, AIC = 1006.2, *p* < 0.001), model 3 was significantly better than model 2 (χ^2^ = 7.01, AIC = 1001.2, *p* < 0.01), and model 4 significantly better than model 3 (χ^2^ = 13.19, AIC = 990.01, *p* < 0.001). In model 4 (see Table 3), age, vocabulary, TCS recognition, perceptual learning, incidental and statistical learning were significant predictors of natural-fast speech recognition. This model suggests that when all other variables are held constant, individuals with better learning skills on each of the learning tasks, have an advantage in natural-fast speech recognition compared to those with poorer learning skills. Thus, for individuals with similar age, memory, vocabulary, statistical and incidental learning, one SD increase in perceptual learning slope is associated with ~53% increase in the odds of correctly recognizing natural-fast speech. Similarly, one SD increase in the incidental and statistical learning is associated with ~20% and ~ 36% (respectively) increase in the odds of correctly recognizing natural-fast speech.

**Table 3:** Estimates of natural-fast speech recognition.

	OR	*β*	SE	*Z*	*P*
Age	1.34	0.30	0.10	2.92	<0.01
Working memory	1.13	0.13	0.08	1.49	0.13
Vocabulary	0.81	−0.20	0.09	−2.20	<0.05
Attention	1.08	0.08	0.09	0.96	0.33
TCS recognition	1.41	0.35	0.09	3.76	<0.001
Perceptual learning	1.53	0.43	0.10	4.26	<0.01
Incidental learning	1.20	0.19	0.08	2.24	<0.05
Statistical learning	1.36	0.31	0.08	3.50	<0.001

#### Recognition of speech in noise

Table 4 shows model 4 for Speech in Noise. Model comparison suggested that model 2 (Basic + perceptual learning) was not significantly different from model 1 (Basic) (χ^2^ = 0.22, AIC = 1,168, *p* > 0.05) nor model 3 was significantly different from model 2 (χ^2^ = 2.61, AIC = 1,167, *p* > 0.05). On the other hand, model 4 fitted the data significantly better than model 3 (χ^2^ = 6.63, AIC = 1,163, *p* < 0.01). Model coefficients are reported in Table 4 which suggests that TCS recognition and statistical learning were the only significant predictor of speech in noise recognition accuracy. According to this model, one SD increase in statistical learning is associated with a ~ 33% increase in the odds of correctly recognizing speech in noise. On the other hand, perceptual and incidental learning were not significant predictors of the recognition of speech in noise.

**Table 4:** Estimates of speech in noise recognition.

	OR	*β*	SE	*Z*	*P*
Age	1.03	0.03	0.12	0.27	0.78
Working memory	0.94	−0.06	0.10	−0.59	0.54
Vocabulary	1.12	0.12	0.11	1.02	0.30
Attention	1	0.00	0.11	0.00	0.99
TCS recognition	1.97	0.68	0.12	5.43	<0.001
Perceptual learning	0.96	−0.04	0.11	−0.42	0.66
Incidental learning	0.16	0.15	0.10	1.46	0.14
Statistical learning	1.33	0.29	0.11	2.66	<0.01

#### Recognition of vocoded speech

Model comparison showed that model 2 (Basic + perceptual learning) did not fit the data better than the Basic model (χ^2^ = 0.006, AIC = 1,072, *p* > 0.05) nor model 3 was significantly different from model 2 (χ^2^ = 2.10, AIC = 1,071, *p* > 0.05). On the other hand, model 4 fitted the data significantly better than model 3 (χ^2^ = 5.85, AIC = 1,068, *p* < 0.05). Table 5 shows the details of model 4. According to this model, age, working memory, vocabulary, and statistical learning were significant predictors of the recognition of vocoded speech. According to this model, one SD increase in statistical learning is associated with a ~ 29% increase in the odds of correctly recognizing vocoded speech.

**Table 5:** Estimates of vocoded speech recognition.

	OR	*β*	SE	*Z*	*P*
Age	1.52	0.42	0.11	3.60	<0.001
Working memory	1.27	0.24	0.10	2.39	<0.05
Vocabulary	0.74	−0.29	0.11	−2.62	<0.01
Attention	1.16	0.15	0.10	1.46	0.14
TCS recognition	1.07	0.07	0.11	0.64	0.51
Perceptual learning	1	0.00	0.10	0.06	0.94
Incidental learning	1.12	0.12	0.10	1.25	0.20
Statistical learning	1.29	0.26	0.10	2.43	<0.05

## Discussion

This study examined the associations between implicit learning and the recognition of challenging speech (natural fast, vocoded and speech in noise). Although associations between individual indices of learning and speech recognition were documented before (Clopper and Pisoni, 2004; Adnak and Janse, 2009; Conway et al., 2010; Vlahou et al., 2012), the simultaneous use of different indices and paradigms of implicit learning (perceptual, statistical and incidental) in the current study made it possible to examine the unique contribution of each index of learning, after accounting for the contribution of both cognitive factors and the other learning indices. Four main findings are noteworthy: (1) Each index of learning had a unique contribution to the recognition of fast speech, consistent with previous studies on perceptual learning (Adnak and Janse, 2009; Manheim et al., 2018; Rotman et al., 2020). (2) Statistical learning predicted the recognition of speech in noise, in line with previous work (Conway et al., 2010). (3) In line with our hypothesis, one index of learning (statistical learning) predicted the recognition of vocoded speech. (4) Of the cognitive factors examined (working memory, vocabulary, and attention), only working memory emerged as a significant predictor of the recognition of vocoded speech. Age was found to be a significant predictor of natural fast speech and vocoded speech but not to speech in noise. “TCS” recognition, which served as a baseline, was found to be a significant predictor to the recognition of natural fast speech and speech in noise but not vocoded speech.

In the current study, all forms of implicit learning had a unique contribution to the recognition of fast speech. Even after accounting for other factors, perceptual learning remained a significant predictor of fast speech. For perceptual learning, this replicates previous findings (Manheim et al., 2018; Rotman et al., 2020) and indicates that rapid perceptual learning on one adverse condition (time-compressed speech) may support speech recognition on a different condition (fast speech). Whereas the contribution of perceptual learning might be questioned because learning was assessed with a speech task, statistical and incidental learning were assessed with visual tasks. Therefore, it appears that the contribution of implicit learning to fast speech recognition extends across sensory modalities. To recognize fast speech, individuals need to “map” the fast, unusual input (Rönnberg et al., 2019, 2021) to stored representations that are based on normal-rate speech. Statistical learning may support pattern recognition. Therefore, our findings suggest that good statistical learning could facilitate the rapid learning of the new mappings, thereby resulting in more accurate recognition of rapid speech. Likewise, incidental learning and fast speech recognition were positively associated in the current study, suggesting that listeners who can improve the processing of features unrelated to the task being practiced might use this to support the recognition of rapidly presented speech.

Of the three learning indices, only statistical learning contributed to the recognition of speech in noise, consistent with the findings of Conway et al. (2010). The common factor involved in both statistical learning speech in noise processing could be an implicit sensitivity to the underlying statistical structure contained in sequential patterns, independent of other cognitive abilities (Conway et al., 2010). Neither perceptual nor incidental learning contributed significantly to the recognition of speech in noise. The insignificant contribution of perceptual learning in the current study is inconsistent with previous demonstrations (Rotman et al., 2020; Banai et al., 2022), even though all studies used similar designs. Given the similarities in talker characteristics, signal to noise ratio and type of noise masker (4-talker babble) across studies, one possibility is that the discrepancy in the results stems from the different number of trials over which learning was assessed (30 here vs. 10 in past studies). If rapid learning supports speech recognition by allowing listeners to rapidly adapt to new auditory challenges (Rotman et al., 2020), perhaps 30 sentences is too long. To the best of our knowledge, no studies evaluated the contribution of incidental learning to the recognition of speech in noise. Therefore, additional studies are required to confirm or refute the current finding.

Consistent with our hypothesis, implicit learning was correlated with vocoded speech, but only for the statistical learning index. To the best of our knowledge, no previous study examined the contribution of implicit learning to the recognition of vocoded speech. A study by Neger et al. (2014) demonstrated an association between statistical learning and learning of vocoded speech. Although the two sets of findings seem similar, we note that their model included an estimate of vocoded speech learning, whereas ours included an estimate of time-compressed speech learning. Thus, the two sets of findings are not directly comparable and further studies are required on the contribution of statistical learning to vocoded speech recognition.

Together, the current findings suggest that perceptual, statistical and incidental learning are independent of each other when it comes to speech recognition. Although modality specific factors could contribute to this disjuncture, they are not likely to fully account for it because here the correlation between learning effects in the two visual tasks were low, whereas a shared factor has been suggested to underlie both auditory and visual perceptual learning following long-term training (Yang et al., 2020). This is contrary to the association between perceptual learning (of vocoded speech) and statistical learning that was reported by Neger et al. (2014). This discrepancy might stem from differences between vocoded speech (a spectral manipulation) and time-compressed speech (a temporal one) or from methodological differences (e.g., longer learning period of 60 sentences in Neger and colleagues; different ways of quantifying learning). Further work is required to determine which is the case. Furthermore, the contribution of each index of learning may depend on the speech task. For example, statistical learning was a significant predictor of natural-fast speech, speech in noise, and vocoded speech, whereas incidental learning was a significant predictor of only natural-fast speech. Although the three indices of implicit learning might share similar neural substrates, each might capture different aspects of the probabilistic nature of the speech input (Fiser and Lengyel, 2019). For example, good perceptual learning could allow listeners to rapidly adapt to the speech characteristics of the talker that produced the fast speech sentences, but not to how segments from each sentence can be ‘glimpsed’ from the noise. On the other hand, good statistical learning could allow listeners to adapt to structures that are common across sentences and thus facilitate the recognition of both fast speech and speech in noise.

Although our focus was on learning, some findings are also relevant to the role of cognitive abilities in speech recognition. Working memory contributed to the recognition of vocoded speech, but not to the recognition of natural fast speech or speech in noise. This finding is in line with previous studies (Davis et al., 2005; Akeroyd, 2008; Hervais-Adelman et al., 2008; Tamati et al., 2013; Neger et al., 2014; Rotman et al., 2020). According to the ELU model, adverse listening conditions can create a mismatch between the degraded signal and the representations of speech which are stored in long-term memory (Rönnberg et al., 2019, 2021). When a mismatch occurs, an explicit processing loop is activated, allowing listeners to decipher the input through explicit working memory processing. In our study, it seems that the mismatch created by vocoded speech may have activated working memory resources to a greater extent than natural fast speech or speech in noise, and therefore an association was evidenced. Perhaps the level of noise was too high for explicit processing to compensate for the loss of sensory detail, consistent with the lower performance in the speech in noise task (see Figure 1). In contrast to working memory, neither attention nor vocabulary significantly contributed to speech recognition in our study, which is consistent with previous reports (Bent et al., 2016; Rotman et al., 2020).

Three main limitations of this study are noteworthy. First, whereas perceptual learning was assessed with an auditory speech task, statistical and incidental learning were assessed with visual tasks. As mentioned above, we used an auditory task because visual perceptual learning is usually not documented within minutes. Nevertheless, the current findings cannot be fully attributed to modality differences because both indices of visual learning were significant contributors to the recognition of fast speech, but auditory perceptual learning was not a significant predictor of speech in noise and vocoded speech recognition. Second, the three speech tasks differed not only in the acoustic manipulation used, but also included different talkers and sentences. Although this could influence the results, perceptual learning, speech in noise and vocoded speech were evaluated with the same talker, yet perceptual learning was not significantly associated with speech recognition. On the other hand, perceptual learning and natural-fast speech recognition were associated even though they were evaluated with different talkers. It therefore seems that talker differences are not a likely explanation of the current findings. As for the use of an unbalanced design with respect to sentences, we note that in each of our previous studies different sentences were used yet the overall pattern of association between speech recognition and rapid learning was consistent across studies (Karawani and Banai, 2017; Rotman et al., 2020; Banai et al., 2022). Third, in the current study, perceptual learning of time-compressed speech was a significant predictor of fast-speech recognition only. Although this could simply reflect the similarities between time-compressed and natural-fast speech, this is not necessarily the case because two previous studies (Rotman et al., 2020; Banai et al., 2022) showed that time-compressed speech learning contributes to speech in noise recognition, but the current study failed to replicate this finding. Nevertheless, using additional learning tasks is advisable for future studies.

To conclude, this study explored the unique contributions of three indices of implicit learning (perceptual, statistical and incidental) to individual differences in the recognition of challenging speech (natural fast, vocoded and speech in noise), beyond the contribution of other known cognitive factors. Findings suggest that although implicit learning contributes to the recognition of challenging speech, the contribution probably depends on the type of speech challenge and on the learning task.

## Data availability statement

The datasets presented in this study can be found in online repositories. The names of the repository/repositories and accession number(s) can be found at: https://osf.io/EY7SG/ (DOI: 10.17605/OSF.IO/EY7SG).

## Ethics statement

All aspects of the study were approved by the ethics committee of the Faculty of Social Welfare and Health Sciences at the University of Haifa (protocol number 419/19). The studies were conducted in accordance with the local legislation and institutional requirements. The participants provided their written informed consent to participate in this study.

## Author contributions

RK and KB designed the study, interpreted the findings, and wrote the manuscript. RK collected and analyzed the data. HK reviewed and edited the manuscript. All authors approved the final version of the manuscript.

## Funding

This study was supported by the Israel Science Foundation grant 206/18.

## Conflict of interest

The authors declare that the research was conducted in the absence of any commercial or financial relationships that could be construed as a potential conflict of interest.

## Publisher’s note

All claims expressed in this article are solely those of the authors and do not necessarily represent those of their affiliated organizations, or those of the publisher, the editors and the reviewers. Any product that may be evaluated in this article, or claim that may be made by its manufacturer, is not guaranteed or endorsed by the publisher.
